# Saikosaponin A Inhibits Triple-Negative Breast Cancer Growth and Metastasis Through Downregulation of CXCR4

**DOI:** 10.3389/fonc.2019.01487

**Published:** 2020-01-28

**Authors:** Ying Wang, Liang Zhao, Xianghui Han, Yahui Wang, Jinxia Mi, Changhong Wang, Duxin Sun, Yunfei Fu, Xiaodong Zhao, Haidong Guo, Qiangli Wang

**Affiliations:** ^1^School of Pharmacy, Shanghai University of Traditional Chinese Medicine, Shanghai, China; ^2^Department of Pharmacy, Shanghai Baoshan Luodian Hospital, Shanghai, China; ^3^Institute of Chinese Traditional Surgery, Longhua Hospital, Shanghai University of Traditional Chinese Medicine, Shanghai, China; ^4^Science and Technology Center, Shanghai University of Traditional Chinese Medicine, Shanghai, China; ^5^Institute of Chinese Materia Medica, Shanghai University of Traditional Chinese Medicine, Shanghai, China; ^6^Department of Pharmaceutical Sciences, College of Pharmacy, University of Michigan, Ann Arbor, MI, United States; ^7^Department of Pathology, National Shanghai Center for New Drug Safety Evaluation and Research, Shanghai, China; ^8^School of Basic Medical Sciences, Shanghai University of Traditional Chinese Medicine, Shanghai, China

**Keywords:** saikosaponin A, natural product, triple-negative breast cancer, metastasis, SDF-1/CXCR4 axis

## Abstract

**Purpose:** Due to a lack of recognized molecular targets for therapy, patients with triple-negative breast cancer (TNBC), unlike other subtypes of breast cancers, generally have not benefited from the advances made with targeted agents. The CXCR4/SDF-1 axis is involved in tumor growth and metastasis of TNBC. Therefore, down-regulation of the expression of CXCR4 in cancer cells is a potential therapeutic strategy for inhibiting primary tumor growth and metastasis of TNBC. In order to identify bioactive compounds that inhibit the expression of CXCR4 in traditional Chinese medicines, we investigated the capacity of saikosaponin A (SSA), one of the active ingredients isolated from Radix bupleuri, to affect CXCR4 expression and function in TNBC cells.

**Methods:** Analyses of cell growth, migration, invasion, and protein expression were performed. Knockdowns by small interfering RNA (siRNA) and non-invasive bioluminescence were also used.

**Results:** SSA reduced proliferation and colony formation of SUM149 and MDA-MB-231 cells. SSA inhibited migration and invasion of TNBC cells. For mice, SSA inhibited primary tumor growth and reduced lung metastasis of highly metastatic, triple-negative 4T1-luc cells. SSA inhibited CXCR4 expression but did not regulate CXCR7 expression *in vitro* and *in vivo*. The inhibitory effects on the migration and invasion of TNBC cells were reversed by down-regulation of CXCR4 expression. In addition, SSA inactivated the Akt/mTOR signaling pathway and inhibited MMP-9 and MMP-2 expression.

**Conclusions:** The results show that SSA exerts an anti-TNBC effect through the inhibition of CXCR4 expression and thus has the potential to be a candidate therapeutic agent for TNBC patients.

## Introduction

Breast cancer is a major risk factor for women's health. In the United States in 2019, it was estimated that 268,600 women would be diagnosed with breast cancer, accounting for 30% of all new cancer diagnoses in women (1). Compared with Western countries, the incidence of breast cancer in China is relatively low. However, with the acceleration of urbanization in China, the incidence of breast cancer has increased rapidly since the 1990s, and the number of cases will reach 2.5 million by 2021 (2). Of all of the subtypes of breast cancer, triple-negative breast cancer (TNBC) has the most lethal characteristics and is associated with a poor prognosis, lacking expression of the estrogen receptor, progesterone receptor, and human epidermal growth factor receptor-2 (HER-2/neu) (3). Due to the lack of defined molecular targets and its biological heterogeneity, there is no approved targeted therapy for TNBC. At present, the main modality of treatment is cytotoxic chemotherapy for patients with either early- or late-stage disease (4). Although there is a relatively high response to cytotoxic chemotherapy, local recurrence in TNBC patients is more likely to cause lung and brain metastases, ultimately leading to death (5).

CXCR4 is involved in tumor growth and metastasis in TNBC (6), implying that there is therapeutic promise for CXCR4-targeted agents (7). CXCR4 binding to its ligand SDF-1 triggers the activation of signaling pathways including phosphoinositide 3-kinase/protein kinase B (PI3k/Akt), ERK1/2, and MAPK (8). TNBC expresses CXCR4 more frequently than other breast cancer subtypes (9), and high expression levels of CXCR4 are related to a high histological grade of TNBC and high incidences of recurrence and cancer-related death (9–11). As a new strategy, targeting CXCR4 or its ligand by antibodies, small peptides, or small interfering RNAs (siRNAs) is effective for inhibiting primary tumor growth and metastasis in TNBC (12–14).

Natural products are a rich source to provide lead compounds for the development of novel drugs (15). Many bioactive compounds from traditional Chinese medicines show anti-tumor effects on TNBC (16–19), and some of these compounds inhibit CXCR4 expression in TNBC cells (19). However, none of the above-cited studies targeted CXCR4 to identify drug candidates against TNBC.

**Figure 1:**
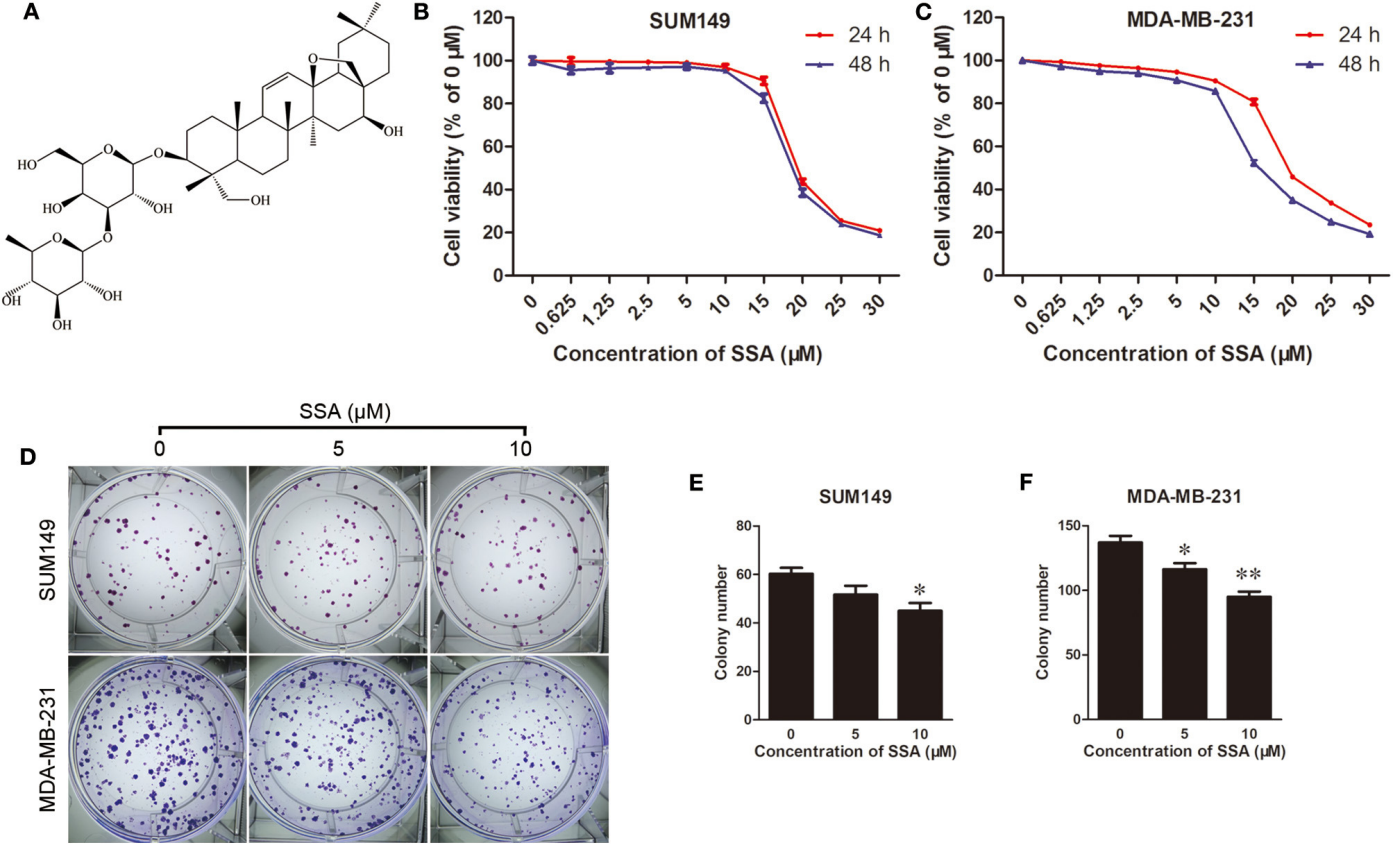
SSA inhibits growth and colony formation of TNBC cells. **(A)** Chemical structure of SSA. SUM149 **(B)** and MDA-MB-231 **(C)** cells were treated with SSA at concentrations of 0, 0.625, 1.25, 2.5, 5, 10, 15, 20, 25, or 30 μM. At 24 and 48 h, cck-8 assays were performed, and cell viability was determined. Data from three independent experiments were pooled. **(D)** SUM149 and MDA-MB-231 cells were treated with SSA at different concentrations of 0, 5, and 10 μM for 12 days. Colonies were stained with crystal violet. Data for SUM149 cells **(E)** and MDA-MB-231 cells **(F)** from three independent experiments were pooled. ^*^p < 0.05; ^**^p < 0.01.

In previous studies (20), we identified saikosaponin A (SSA), which possesses the potential to inhibit CXCR4 expression, from 100 herbal monomers via dual-luciferase assay. In the present study, the inhibitory effects of SSA on the expression of CXCR4 and growth of TNBC cells in culture and in mice were investigated. SSA is a triterpenoid glycoside (Figure 1A), a primary active component of the plant Radix bupleuri. SSA has anti-inflammatory, anti-fibrotic, anti-epileptic, analgesic, neuro-modulatory, and anti-cancer activity (21). SSA inhibits human breast cancer cell proliferation and induces apoptosis (22). However, it is unclear whether SSA down-regulates CXCR4 expression in TNBC. Here, we confirmed that SSA is a potent inhibitor of CXCR4 and that it suppressed tumor growth and metastasis of TNBC through its effects on the CXCR4/SDF-1 axis.

## Methods

### Cell Cultures

The human SUM149 and MDA-MB-231 cell lines (TNBC) and murine 4T1-luc cells (TNBC, highly metastatic) were obtained from Cobioer (Nanjing, China). SUM149 cells were maintained in Ham's F12 medium supplemented with 5% (v/v) fetal bovine serum (FBS), 5 μg/mL of insulin, 1 μg/mL of hydrocortisone, 100 U/mL of penicillin, and 100 μg/mL of streptomycin. MDA-MB- 231 cells were maintained in Dulbecco's Modified Eagle Medium (DMEM) supplemented with 10% (v/v) FBS, 100 U/mL penicillin, and 100 mg/L streptomycin. The 4T1-luc cells were maintained in RPMI 1640 medium supplemented with 10% (v/v) FBS, 100 U/mL penicillin, and 100 mg/L streptomycin. The cells were incubated at 37°C in a 5% CO_2_ incubator with saturated humidity.

### Cell Proliferation Assays

SSA was purchased from Shanghai Winherb Medical Technology Co., Ltd. (Shanghai, China). SUM149 and MAD-MB-231 cells were seeded into 96-well plates separately. Twelve hours later, the culture medium was replaced with fresh medium containing various concentrations of SSA (0, 0.625, 1.25, 2.5, 5, 10, 15, 20, 25, and 30 μM). After incubation for 24 or 48 h, cell viability was assessed by the use of a Cell Counting Kit-8 (CCK-8; Dojindo Laboratories, Kumamoto, Japan) according to the manufacturer's protocol. Briefly, the medium was replaced by 100 μl of fresh medium containing 10 μl of CCK-8 solution, and cells were incubated for another 2 h. Values of OD450 nm were measured with a BioTek instrument (Winooski, Vermont, USA).

### Colony Assays

To further determine the inhibitory effect of SSA on the tumorigenicity of TNBC cells, colony formation assays were performed. Two hundred SUM149 or MDA-MB-231 cells were seeded into 6-well plates to incubate overnight. The cells were then incubated with different final concentrations (0, 5, and 10 μM) of SSA for 10–15 days. After fixing with 4% paraformaldehyde and staining with a crystal violet solution, colonies containing more than 50 individual cells were counted under a stereomicroscope.

### Animal Preparation and Tumor Xenografts

The study was approved by the Ethics Committee of Shanghai University of Traditional Chinese Medicine and conducted in accordance with the principles of the Basel Declaration and the recommendations of the Guidelines for the Care and Use of Laboratory Animals. Seven-week-old female athymic BALB/c nude mice (SLAC Laboratory Animal Co., Ltd., Shanghai, China) and BALB/c mice (Charles River Laboratories, Beijing, China) were raised under specific-pathogen-free environmental conditions at an animal experiment center at the Shanghai University of Traditional Chinese Medicine. One million MDA-MB-231-Luc cells were suspended in 0.1 mL of a mixture (equal volumes) of PBS and Matrigel (Corning, New York, USA) and injected orthotopically into the upper left mammary fat pads of nude mice. When the tumor volume reached ~100 mm^3^, the mice were randomly assigned into four groups for treatment: control (*n* = 5); paclitaxel (*n* = 5); AMD3100, a CXCR4 antagonist (*n* = 5); and SSA (*n* = 5). Experiments were then performed. Tumor volumes were calculated with the formula V= (π/8) a × b^2^, where V denotes tumor volume, a denotes maximum tumor diameter, and b denotes minimum tumor diameter. To assess the overall health of the animals, body weight was measured once a week as an indicator.

To assess the effect of SSA on the metastasis of TNBC cells, 1 × 10^5^ 4T1-luc cells suspended in 0.1 mL of PBS were injected into the tail veins of BALB/c mice. The mice were then randomly assigned to the four groups detailed above.

### Therapies

The animals in the orthotopic breast tumor models were treated when the tumor volume reached ~100 mm^3^ as follows: (1) mice in the control group were intraperitoneally injected twice a week with the vehicle, a mixture of equal volumes of Cremophor El and ethanol, (2) mice in the paclitaxel group were intraperitoneally injected with paclitaxel (30 mg/kg) dissolved in the vehicle once a week, (3) mice in the AMD3100 group were intraperitoneally injected every day with AMD3100 (2.5 mg/kg) dissolved in PBS, and (4) mice of the SSA group were intraperitoneally injected once a week with SSA (12 mg/kg) in the vehicle. The volume of each intraperitoneal injection was 0.1 mL. Therapies were administered until the tumor volumes reached 2,000 mm^3^, and animals were sacrificed by CO_2_ asphyxiation. The animals used in the lung metastasis model were treated 2 h after transplantation and sacrificed by euthanasia 2 weeks later.

### Bioluminescence Imaging

For nude mice engrafted with MDA-MB-231-Luc cells, whole-body bioluminescence imaging was performed to obtain baselines at the beginning of therapy. Tumor volumes were examined weekly using the IVIS system (Xenogen, Alameda, CA, USA). Mice were intraperitoneally injected with 150 mg/kg of D-luciferin dissolved in PBS (15 mg/mL) and then anesthetized with isoflurane. At 10 min after the injection, bioluminescence images were collected for 1–60 s, depending on the amount of luciferase activity. Bioluminescence from regions of interest (ROI) was defined manually around the mammary fat pad tumor, and data were expressed as photon flux (photons/s/cm^2^/steradian) using Living Image software (version 2.50, Xenogen, Alameda, CA, USA). Metastases to the lung were similarly quantified at day 14 after implantation of 4T1-luc.

### Histological Analysis of Lung Metastasis

To observe the lung metastasis of 4T1-luc cells, the lungs of each group were fixed with 4% paraformaldehyde and embedded in paraffin 14 days after tail vein injection. After being cut into 5 μm-thick sections, the lung tissues were stained with hematoxylin–eosin (H&E) and then examined under a stereomicroscope or upright optical microscope (100 magnification).

### Plasmid Construction

A 279-bp sequence from the human CXCR4 promoter (−191 to +88) (23) was inserted into the *XhoI*/*Hind III* site of the pGL4.17 plasmid, which contains the firefly luciferase gene (YouBio, Hunan, China). The recombinant plasmid, confirmed by sequencing, was named pGL4.17- CXCR4p.

### Transient Transfection and Luciferase Assay

pGL4.17-CXCR4p was transfected into Human Embryonic Kidney 293 (HEK-293) cells using SuperFectin II DNA Transfection Reagent (Pufei Biotech, Shanghai, China) according to the manufacturer's instructions. A pRL-TK plasmid containing the Renilla luciferase gene (YouBio, Hunan, China) was co-transfected as an internal control. The transfection was accomplished in 24-well plates; the final concentrations of pGL4.17-CXCR4p and pRL-TK were 0.65 and 0.35 μg/mL, respectively. After G418 resistance screening and single-cell clone culture, positive cells were used for luciferase assays in triplicate in 96-well plates by use of luciferase assay kits (Promega, Madison, WI) according to the manufacturer's protocol.

### Wound-Healing Assay

SUM149 and MAD-MB-231 cells were seeded into 6-well culture plates at a density of 5.0 × 10^5^ cells per well and cultured for 24 h to grow to 90% confluency in complete medium. Then the cells had been serum-starved for 24 h, and monolayers of confluent cells were scraped with a 200-μl sterile pipette tip to obtain a wound in each well. After removing the floating cells by washing the scraped surface three times with PBS, wounded monolayers were photographed with a microscope. Cells were then incubated in medium in the absence or presence of SSA (0, 2.5, 5, or 10 μM) for 24 h. After incubation, the medium containing SSA was changed to complete medium containing 100 ng/mL SDF-1α. The images of cells migrating into the wound surface and the average distance of migrating cells were determined under a phase-contrast microscope at 24 (for MDA-MB-231 cells) or 48 (for SUM149 cells) h later.

### Transwell Migration and Invasion Assay

To evaluate cell migration, the upper chambers of Transwell migration chambers with 8-μm pores (Falcon, New York, USA) were seeded with cancer cells (1 × 10^5^). Twelve hours later, the culture medium was changed to serum-free medium containing SSA at concentrations of 0, 2.5, 5, or 10 μM. After incubation for 24 h, 600 μl of complete medium containing 100 ng/mL SDF-1α was added to the lower chambers. Cells were then allowed to migrate for 24 h. The upper surfaces of the chambers were wiped with a cotton swab, and migrated cells were fixed with 3.5% paraformaldehyde in PBS then stained with 0.2% crystal violet (Beyotime Biotechnology, Nantong, China). The numbers of invading cells were counted in five randomly selected microscope fields (×100). To evaluate the invasion of cells, the BD Bio-Coat Matrigel invasion assay system (BD Biosciences, CA, USA) was used according to the manufacturer's instructions. The conditions for invasion assays were the same as for the Transwell assays.

### siRNA Analysis

A double-stranded siRNA against CXCR4 was synthesized by GenePharma (Shanghai, China). The sequences were sense, 5′CUGUCCUGCUAUUGCAUUATT3′ and anti-sense, 5′UAAUGCAAU AGCAGGACAGTT3′. Negative control siRNA sequences were sense, 5′ UUCUCCGAACGU GUCACGUTT3′ and antisense, 5′ACGUGA CACGUUCGGAGAATT3′. For transfection, SUM149 and MDA-MB-231 cells were seeded in 6-well plates for 12 h to produce 90–95% confluence. Cells were then transfected with the siRNA against CXCR4 for 24 h at a concentration of 40 nM with Hieff Trans^TM^ Liposomal Transfection Reagent (Yeasen, Shanghai, China) according to the manufacturer's instructions. Transfection with a control siRNA served as a negative control. To measure the effects of the siRNA, cells were subjected to Western blotting to detect CXCR4 expression after incubation in complete medium for 24 h. The transfected cells were seeded for wound-healing, Transwell migration, and invasion assays.

### Western Blotting Analysis

After SUM149 and MDA-MB-231 cells were treated with SSA (0, 2.5, 5, or 10 μM) for 24 h, total cell protein lysates were extracted using RIPA lysis buffer that contained a protease inhibitor cocktail and a phosphatase inhibitor cocktail. Protein lysates (40 μg), determined by BCA analysis (Beyotime, Shanghai, China), were then subjected to Western blotting analysis. The primary antibodies used in analyses were as follows: CXCR4 (1:1,000, Novus, USA), CXCR7 (1:1,000, Proteintech, China), AKT (1:500, Cell Signaling Technology, USA), p-AKT (1:2,000, Cell Signaling Technology, USA), mammalian target of rapamycin (mTOR, 1:1,000, Proteintech, China), matrix metalloproteinase-9 (MMP9, 1:500, Cell Signaling Technology, USA), MMP2 (1:500, Cell Signaling Technology, USA), and caspase 3 (1:1,000, Proteintech, China). The second antibody was a goat anti-rabbit secondary antibody coupled to horseradish peroxidase (1:20,000, Proteintech, China).

To further investigate the effect of SSA on the expression of these proteins *in vivo*, lungs from each group were collected and subjected to Western blotting as described above.

### Immunohistochemical Staining

To evaluate the expression of CXCR4 in TNBC cells transplanted orthotopically (MDA-MB-231-luc) or migrated to lung (4T1-luc), immunohistochemical staining was performed by using VECTASTAIN ABC kits (VECTOR, CA, USA). Briefly, specimens were embedded in Tissue-Tek OCT Compound (Sakura Finetek, CA, USA) and cut into 5 μm sections. After incubation in 3% hydrogen peroxide and blocking serum, the sections were reacted with primary antibody (1:100, Novus, USA) overnight at 4°C in humidified chambers. The sections were then incubated in a biotinylated secondary antibody for 30 min at room temperature after washing three times with PBS. The sections were stained with a complex of avidin and biotinylated horseradish peroxidase (HRP) for 30 min, followed by diaminobenzidine (DAB) staining. After counterstaining with hematoxylin, the sections were examined under a light microscope.

### Statistical Analysis

Data were expressed as mean ± SEM, and *P* < 0.05 was considered statistically significant. Statistical analyses were performed using Student's *t* test and one-way analysis of variance (ANOVA) with the use of SPSS 18.0.

## Results

### SSA Reduces Proliferation and Colony Formation of TNBC Cells

The effects of SSA on the proliferation of TNBC cells were evaluated with CCK-8 kits. SSA inhibited the proliferation of SUM149 and MDA-MB-231 cells in a concentration-dependent manner. After these cells were incubated with SSA for 24 or 48 h, the IC50 values for SUM149 cells were 19.18 ± 0.99 and 19.54 ± 1.28 μM (*n* = 3), respectively, and the IC50 values for MDA-MB-231 cells were 18.26 ± 1.20 and 16.26 ± 0.25 μM (*n* = 3), respectively (Figures 1B,C). Therefore, the lower doses of SSA (0, 2.5, 5, 10 μM) were selected for the following studies. To exclude deviations in the results of the subsequent experiments that might be caused by the cytotoxicity of low-dose SSA, flow cytometry (Data Sheet 1) was performed after staining the cells with Annexin V-FITC/propidium iodide (PI) (BD, USA). The results showed no significant difference in the percentage of apoptotic cells between the control (0 μM SSA) and the lower doses of SSA (5, 10 μM) (Supplementary Figure 1A). To further confirm this, the protein levels of caspase 3 were evaluated by Western blotting, and similar results were obtained (Supplementary Figure 1B). Next, the inhibitory effects of lower doses of SSA (5, 10 μM) on the colony formation of TNBC cells were examined. After 12 days of continuous culture, SSA also reduced the colony formation of SUM149 and MDA-MB-231 (Figures 1D–F). These findings suggested that SSA had potential anti-TNBC properties.

### SSA Inhibits Tumor Growth *in situ*

**Figure 2:**
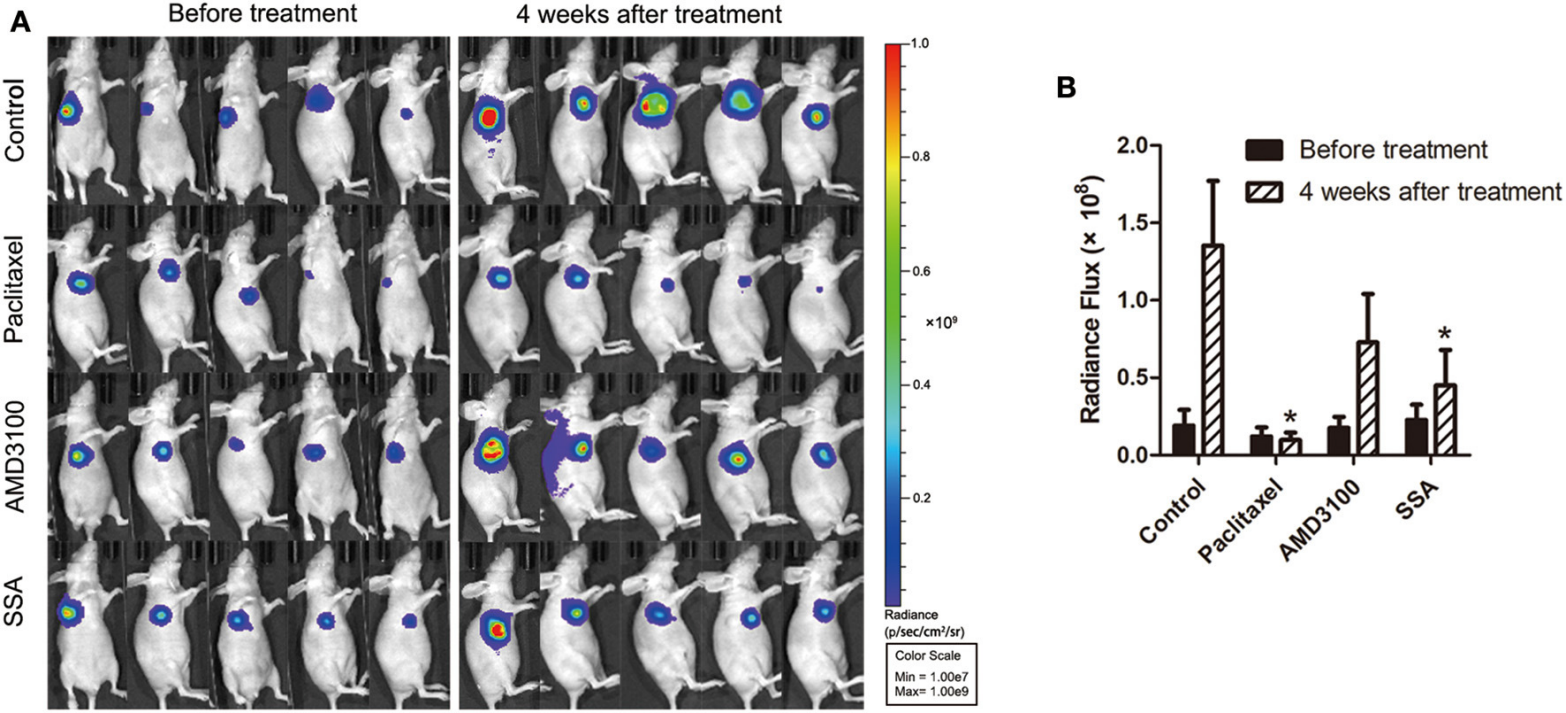
SSA reduces orthotopic tumor growth of MDA-MB-231-luc cells. **(A)** Luciferase imaging of primary tumors before and 4 weeks after treatment with paclitaxel, AMD3100, or SSA. **(B)** ROI analysis of images was used to quantify bioluminescence from orthotopic tumors of mice. The data represent mean values ± SEM for photon flux in each group (*n* = 5). ^*^p < 0.05, vs. control.

To investigate the effect of SSA in inhibiting tumor growth in animals, athymic BALB/c nude mice bearing MDA-MB-231-luc cells were injected intraperitoneally with 12 mg/kg of SSA weekly after the establishment of orthotopic breast cancer tumors. Bioluminescent imaging demonstrated that SSA suppressed primary tumor growth compared to that in control mice (Figure 2A). At the 4-week time point, the mice treated with SSA had a 3-fold reduction in photon flux [(0.45 ± 0.23) × 10^8^ vs. (1.35 ± 0.42) × 10^8^, *p* < 0.05] in primary tumor burden compared to that in the control mice. The photon flux values for the paclitaxel group and the AMD3100 group were (0.10 ± 0.04) × 10^8^ and (0.73 ± 0.31) × 10^8^, respectively (Figure 2B).

### SSA Inhibits Migration and Invasion of TNBC Cells Toward SDF-1α and Lung Metastasis of 4T1 in Mice

**Figure 3:**
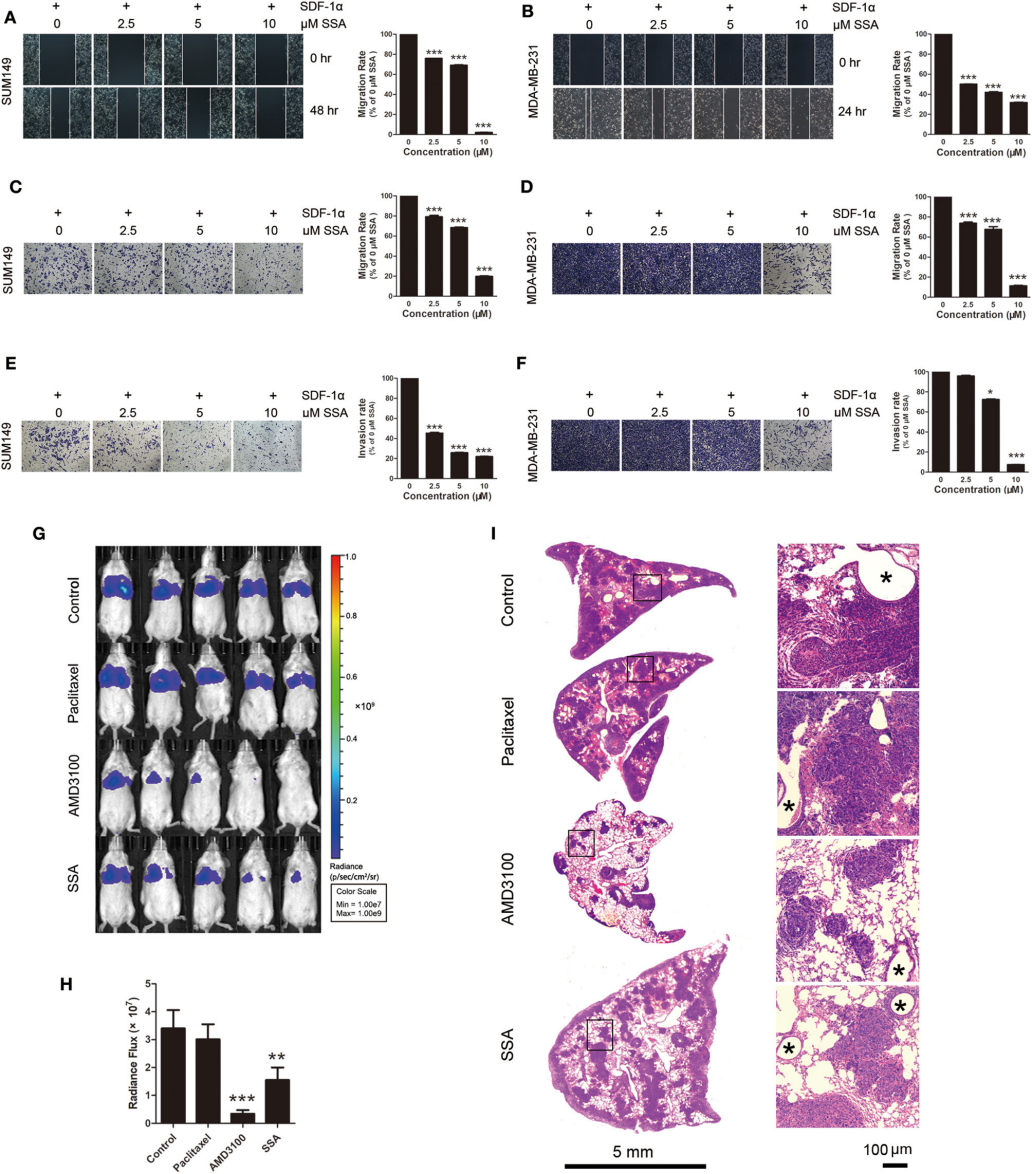
SSA suppresses the migration and invasion of TNBC cells and the lung metastasis of 4T1-luc cells. **(A,B)** Photographs and quantification of wounds to SUM149 and MDA-MB/231 cells treated with SSA. **(C,D)** Photographs and quantification of SUM149 and MDA-MB/231 cell migration through Transwell migration chambers with 8-μm pores and stained with 0.2% crystal violet. **(E,F)** Photographs and quantification of SUM149 and MDA-MB/231 cell invasion through Matrigel-coated polycarbonate membranes, with cells stained by 0.2% crystal violet. The independent experiments were performed in triplicate. ^*^p < 0.05; ^***^p < 0.001 compared with 0 μM of SSA. **(G)** Luciferase imaging of lung metastases at 2 weeks after injection of 4T1-luc cells and treatment with paclitaxel, AMD3100, or SSA. **(H)** ROI analysis of images obtained at 2 weeks was used to quantify changes in cell numbers in lungs after treatment with paclitaxel, AMD3100, or SSA. The data represent mean values ± SEM for photon flux in each group (*n* = 5). ^**^p <0.01, ^***^p <0.001 vs. control. **(I)** H&E staining of lung tissue of 4T1-luc mice. The photographs in the right row are magnifications of the boxes in the left row. Asterisks indicate bronchioles in lung tissue.

To assess the capacity of SSA to inhibit migration of SUM149 and MDA-MB-231 cells, wound scratch healing and Transwell migration assays were carried out. Migration from the extracellular membrane is a first step in the metastasis of cancer cells (24). Tumor cells were incubated with SDF-1α (100 ng/mL) after treatment with SSA. The cells exposed to 0 μM SSA showed a relatively high degree of wound closure (Figures 3A,B) and migration through Transwell membranes (Figures 3C,D), although effects were diminished in the SSA-treated groups in a concentration-dependent manner. The results of Matrigel invasion assays showed that SSA inhibited cell invasion in a concentration-dependent manner (Figures 3E,F).

To determine if SSA inhibits the metastasis of TNBC cells *in vivo*, BALB/c mice injected with 4T1-luc cells through the tail vein were dosed with SSA as described above. At 2 weeks after transplantation of 4T1-luc cells, evaluation of the bioluminescent signals for the lungs revealed less metastasis for SSA-treated mice compared to control mice [photon flux (1.55 ± 0.43) × 10^7^ vs. (3.40 ± 0.66) × 10^7^, *p* < 0.01]. The photon fluxes for the groups dosed with paclitaxel or AMD3100 were (3.01 ± 0.54) × 10^7^ and (0.34±0.13) × 10^7^, respectively (Figures 3G,H).

To further confirm the inhibitory effects of SSA on lung metastasis of 4T1-luc cells, lung tissues were sectioned and subjected to H&E staining after sacrifice of mice. The numbers and sizes of tumors were significantly reduced in the SSA and AMD3100 groups as compared to the control group, but no obvious changes were observed in the paclitaxel group (Figure 3I).

### CXCR4 Is a Target of SSA in Inhibiting Metastasis of TNBC Cells

**Figure 4:**
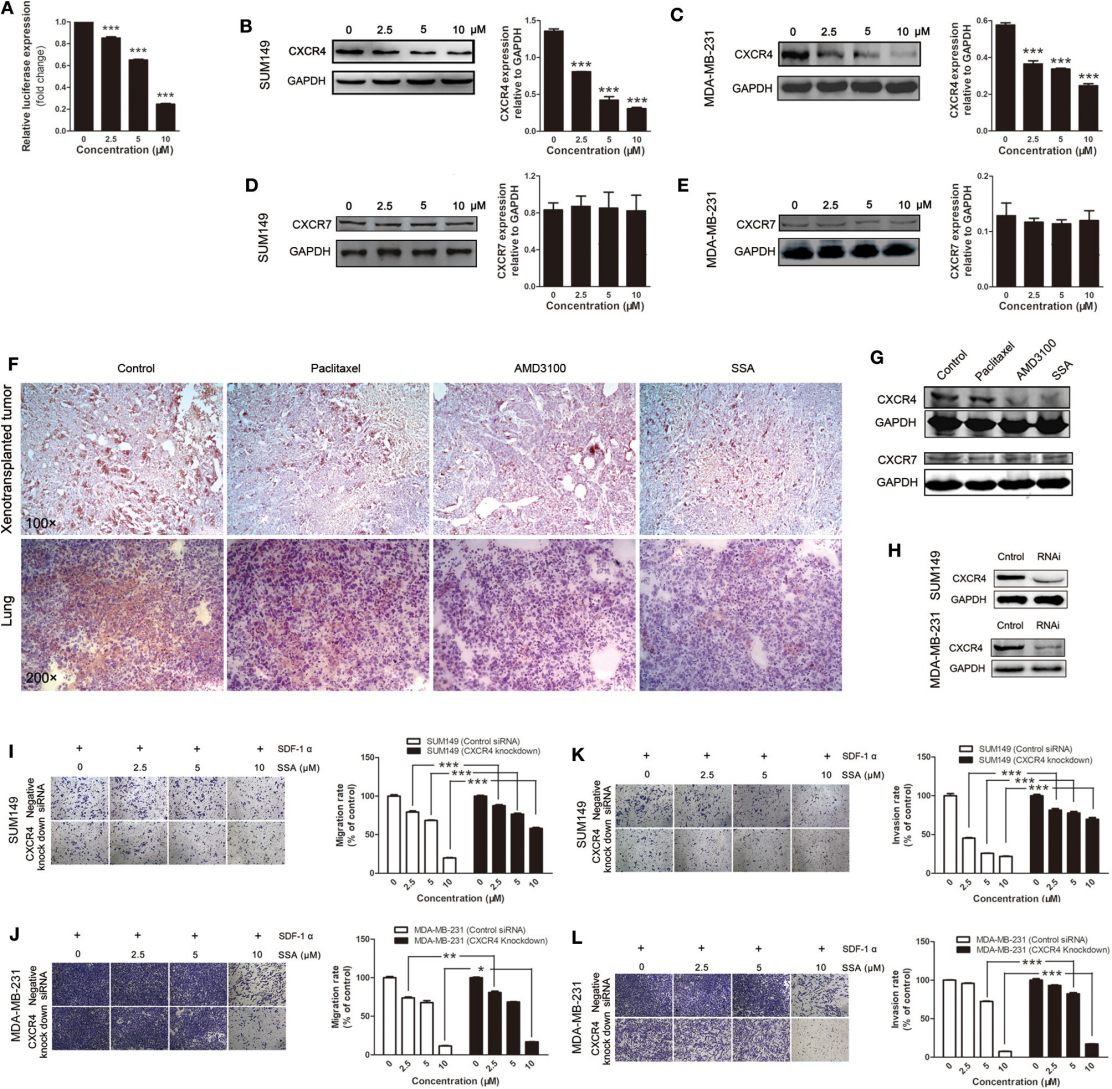
SSA down-regulates CXCR4 to inhibit the migration and invasion of TNBC cells. **(A)** SSA inactivates the CXCR4 promoter in a concentration-dependent manner. SUM149 **(B)** and MDA-MB-231 **(C)** cells were incubated with SSA at various concentrations for 24 h. CXCR4 expression of cells was analyzed by Western blot. The histograms on the right of Western blots show the expression levels of CXCR4 in SUM149 and MDA-MB-231 cells. Data from three independent experiments were pooled. ^***^p < 0.001 vs. 0 μM of SSA. CXCR7 protein expression of SUM149 **(D)** and MDA-MB-231 **(E)** after incubating with SSA at various concentrations for 24 h. The histograms on the right of Western blots show the expression levels of CXCR7 in SUM149 and MDA-MB-231 cells. Data from three independent experiments were pooled. **(F)** Sections of xenografts (upper row) and lung tissue (lower row) in different groups were submitted to immunohistochemistry using antibodies of CXCR4. **(G)** Western blot analysis detection of the CXCR4 and CXCR7 protein expression in the lung tissue of 4T1-luc mice in different groups. **(H)** Western blot analysis of CXCR4 protein expression in SUM149 and MDA-MB-231 cells transfected with an siRNA against CXCR4 for 24 h. SUM149 and MDA-MB/231 cells were transfected with an siRNA against CXCR4 followed by treatment with SSA for 24 h at the indicated concentrations. Transwell **(I,J)** and invasion **(K,L)** assays were performed. The independent experiments were performed in triplicate. ^*^p < 0.05; ^**^p < 0.01; ^***^p < 0.001 compared with cells transfected with control siRNA.

Dual-luciferase assay was performed to evaluate the effects of SSA on the activities of the CXCR4 promoter; results were expressed as relative light units. There were inhibitory effects for 2.5, 5, and 10 μM of SSA, and there was a concentration-response effect (Figure 4A). Subsequently, Western blotting analysis was applied to quantify the expression of CXCR4 in SUM149 and MDA-MB-231 cells. The results showed that SSA suppressed the expression of CXCR4. At concentrations of 2.5, 5, and 10 μM, the levels of CXCR4 expression were down-regulated in a concentration-dependent manner (Figures 4B,C). CXCR7 is another receptor for SDF-1. By binding to SDF-1, CXCR7 participates in tumor growth and metastasis of TNBC as CXCR4 (25). To verify whether the inhibitory effect of SSA on CXCR7 in TNBC cells was similar to that of CXCR4, Western blotting analysis was performed. Unexpectedly, the results indicated that SSA cannot down-regulate the expression of CXCR7 in TNBC cells (Figures 4D,E).

To further confirm the inhibitory effects of SSA on CXCR4 expression *in vivo*, immunohistochemical analysis was performed in xenograft tumors and lung tissues. The results showed that, like AMD3100, SSA significantly reduced the expression of CXCR4 in tumors transplanted *in situ* or migrated to the lung (Figure 4F). Similar results were obtained in Western blotting analysis (Figure 4G). However, paclitaxel was observed to have relatively weak inhibitory effects of on CXCR4 expression in both immunohistochemistry and Western blotting analysis (Figures 4F,G). We also examined the expression of CXCR7 in lung tissue in animals that were injected 4T1-luc cells via the tail vein, but no significant changes were observed (Figure 4G).

To verify the role of CXCR4 in SSA-induced inhibition of cell migration and invasion, siRNA was used to knock down CXCR4 in SUM149 and MDA-MB-231 cells (Figure 4H), and then experiments similar to those described above were performed. The results showed that when CXCR4 expression in TNBC cells was downregulated, the inhibitory effects of SSA on migration through Transwell membranes (Figures 4I,J) and invasion (Figures 4K,L) were attenuated. It is suggested that CXCR4 is the main factor through which SSA inhibits cell migration and invasion.

### SSA Suppresses the Expression of Phospho-Akt, mTOR, MMP-2, and MMP-9 in TNBC

**Figure 5:**
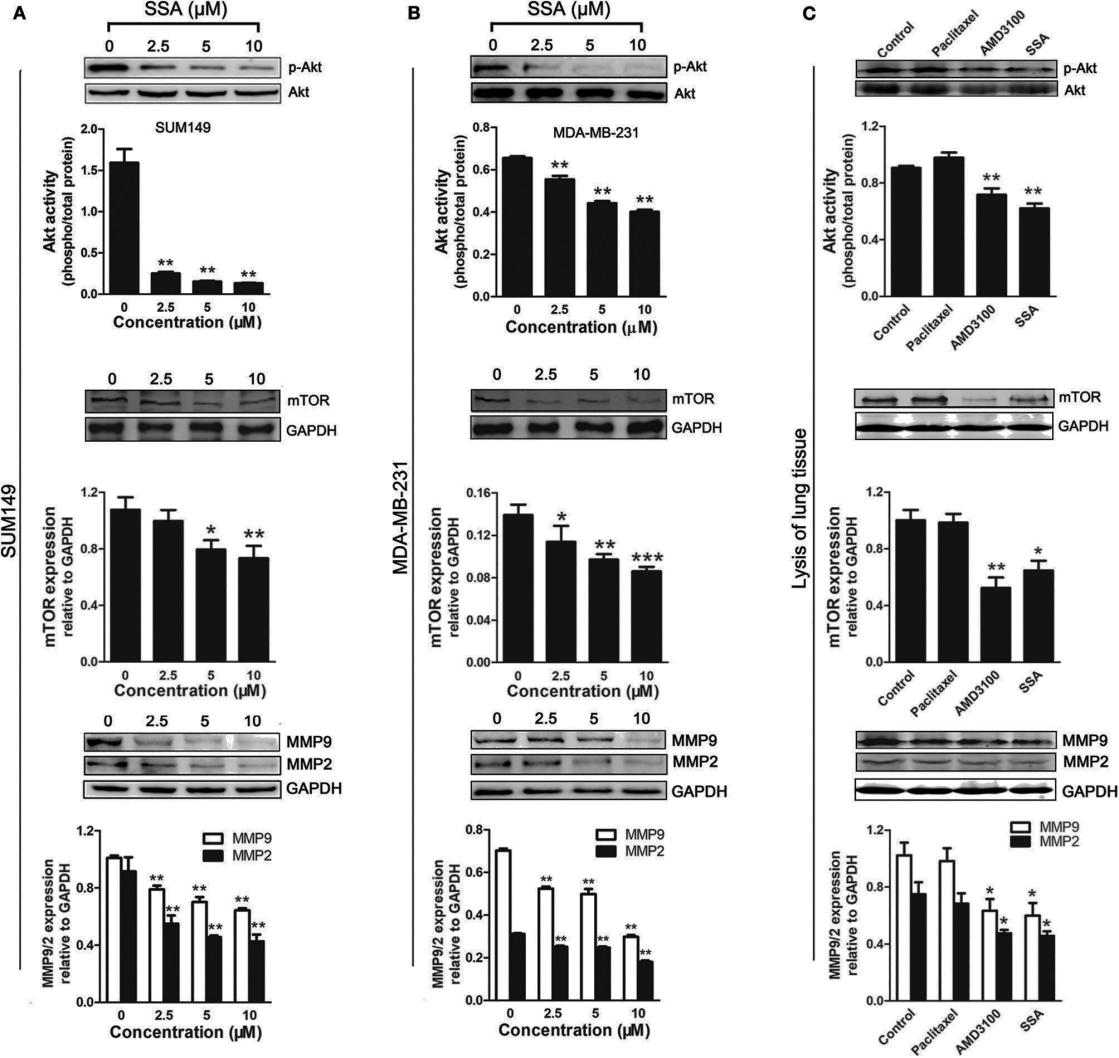
Effect of SSA on the expression of phospho-Akt, mTOR, MMP-2, and MMP-9 in TNBC cells and lung tissue of 4T1-luc mice. Western blot analysis of phospho-Akt, mTOR, MMP-2, and MMP-9 expression in SUM149 **(A)** and MDA-MB-231 **(B)** cells after treatment with SSA at the indicated concentrations for 24 h. Quantification of protein expression is shown below the Western blots. ^*^p < 0.05; ^**^p < 0.01; ^***^p < 0.001 compared with 0 μM of SSA. **(C)** Western blot analysis of phospho-Akt, mTOR, MMP-2, and MMP-9 expression in lung tissue of 4t1-luc mice after treatment with paclitaxel, AMD3100, and SSA for 2 weeks. Quantifications of C are shown below the Western blots. The independent experiments were performed in triplicate. ^*^p < 0.05; ^**^p < 0.01 compared with control group.

The PI3K/Akt signaling pathway is activated by the binding of CXCR4 and SDF-1α (26, 27). mTOR is a downstream effector of the PI3K/Akt axis (28), and MMPs are regulated by CXCR4 in tumor metastasis through the PI3K/Akt signaling pathway (29). To investigate the potential mechanisms by which SSA inhibits the proliferation and migration of TNBC cells, Western blotting analyses were, respectively, used to determine the expression of phospho-Akt, mTOR, MMP-2, and MMP-9 in SUM149 (Figure 5A) and MDA-MB-231 (Figure 5B) cells, following the down-regulation of CXCR4 by SSA. SSA downregulated the expression of phospho-Akt, mTOR, MMP-2, and MMP-9 in a concentration-dependent manner, suggesting that SSA suppressed the downstream targets of CXCR4, including mTOR, MMP-2, and MMP-9, via the PI3K/Akt pathway.

To further verify the down-regulation of these proteins after treatment *in vivo*, the lung tissues of 4T1-injected mice were lysed and subjected to Western blot analysis. Similar inhibitory effects on phosphorylated Akt, mTOR, MMP9, and MMP2 expression were observed in the SSA and AMD3100 groups, but not in the paclitaxel group (Figure 5C).

## Discussion

We established that SSA inhibited the growth of TNBC cells in culture and in mice. Furthermore, we found that SSA suppressed the metastasis of TNBC cells. To elucidate the molecular mechanism by which SSA inhibits the growth and metastasis of TNBC, the CXCR4/SDF-1 axis and its downstream signaling pathways were investigated. CXCR4/SDF-1 axis plays a vital role in breast cancer growth and metastasis (30). In TNBC, there is high expression of CXCR4, and down-regulation of CXCR4 expression inhibits TNBC growth and metastasis (7, 14). Therefore, CXCR4 is a potential therapeutic target for the diagnosis and therapy of TNBC. In our study, it was found that SSA inhibited the activity of the CXCR4 promoter and downregulated CXCR4 expression levels *in vitro* and *in vivo*. To determine whether CXCR4 was a target of SSA in inhibiting the metastasis of TNBC cells, we applied an siRNA to knock down CXCR4 in TNBC cells. After knockdown of CXCR4, the effects of SSA on the inhibition of migration and invasion were reduced. Similar to CXCR4, CXCR7 binding to SDF-1 is also involved in regulating breast cancer growth and metastasis (25). Therefore, we investigated the effects of SSA on CXCR7 expression *in vitro* and *in vivo*. However, no significant differences were observed. These results demonstrate that SSA suppresses the growth and metastasis of TNBC cells through action on CXCR4.

Signaling through CXCR4 activates the PI3K/AKT pathway (26), which is involved in the initiation and progression of breast tumors and regulates various cellular functions, including survival, proliferation, and metabolism (31, 32). In TNBC, the oncogenic PI3K/AKT pathway is activated by overexpression of epidermal growth factor receptor (EGFR) (one of the upstream regulators), dysfunction of phosphatase and tensin homolog (PTEN), and mutations of PI3K catalytic subunit α (PIK3CA). Akt is an integrator of these upstream inputs, and its phosphorylation is a trigger for the activation of the PI3K signaling pathway. We confirmed that SSA downregulated the phosphorylation of Akt (p-AKT) *in vitro* and *in vivo*, suggesting that inhibition of the PI3K/Akt signaling pathway was a step following inhibition of CXCR4 by SSA. In addition, we observed that paclitaxel, unlike SSA, increased p-AKT expression in lung tissue and exhibited lower inhibiting efficacy on TNBC lung metastasis than did SSA in the 4T1 mouse model. Wen et al. (33) also reported the up-regulation effect of paclitaxel on p-AKT, which suggests a possible mechanism for the relatively poor overall survival of patients with metastatic TNBC treated with paclitaxel (34).

In recent years, many cancer studies have described the crosstalk between the CXCR4/SDF-1 axis and the Akt/mTOR pathway and demonstrated the inhibitory effect of CXCR4 antagonist (AMD3100) on mTOR expression (35). We found that SSA inhibited mTOR expression *in vitro* and *in vivo*, suggesting that mTOR inhibition is downstream of the CXCR4 axis. Similar results were obtained in the 4T1-luc mice treated with AMD3100. However, an inhibitory effect on mTOR was not observed in mice treated with paclitaxel. This may be a potential mechanism through which paclitaxel shows lower anti-TNBC metastasis capability. Similar results were reported by Wen et al. (33).

MMPs, which are highly expressed in tumor tissues, degrade the extracellular matrix, a process necessary for invasion and metastasis of breast cancer cells (36). MMP9 and MMP2 are proteases in the MMP family, which, in TNBC tissues, are necessary for extracellular matrix remodeling and cancer cell invasion. CXCR4 is an essential mediator of MMP2 expression and activation (37). Inhibition of MMP9 and MMP2 restricts the capacity of TNBC cells for invasion and metastasis (38). In our study, the levels of MMP9 and MMP2 in TNBC cells were significantly downregulated by SSA, suggesting that their inhibition in TNBC cells was a mechanism by which SSA inhibited the invasion and metastasis of TNBC.

Our study demonstrated that SSA inhibits the proliferation, invasion, and metastasis of TNBC in both cultured cells and mice. By downregulating the expression in TNBC cells of CXCR4, which is involved in the PI3K/Akt/mTOR and MMP signaling pathways, SSA demonstrated anti-growth and anti-metastasis effects on TNBC. However, due to experimental limitations, a direct inhibitory effect of SSA on these signaling pathways, instead of triggered by CXCR4, cannot be ruled out, so the inhibitory effect of SSA on TNBC may involve multiple signaling pathways besides the CXCR4/SDA-1 axis. These results indicate that SSA can be used as a CXCR4 inhibitor and is a candidate therapeutic agent for TNBC patients.

## Data Availability Statement

The datasets used and/or analyzed during the current study are available from the corresponding author on reasonable request.

## Ethics Statement

The animal study was reviewed and approved by Shanghai University of Traditional Chinese Medicine Ethics Committee on the Use of Live Animals for Teaching and Research.

## Author Contributions

YiW, QW, and HG made substantial contributions to the research design, and/or to the acquisition, analysis, and interpretation of data. CW and DS provided substantial contributions to the conception and design of the study. YiW, LZ, and XH completed the luciferase, proliferation, Colony, wound healing, Transwell, and invasion assays. QW, YaW, and YF performed tumor xenografts and therapies of mice and Western blot experiments. JM performed the bioluminescence imaging assay of mice. XZ performed histological analysis and immunohistochemical staining. YiW and QW drafted the manuscript. All authors read and approved the final manuscript.

### Conflict of Interest

The authors declare that the research was conducted in the absence of any commercial or financial relationships that could be construed as a potential conflict of interest.
