# Supplementary material for: Saikosaponin A Inhibits Triple-Negative Breast Cancer Growth and Metastasis Through Downregulation of CXCR4
Source: Front Oncol. 2020 Jan 28;9:1487. doi: 10.3389/fonc.2019.01487 (PMC6997291; doi:10.3389/fonc.2019.01487)
Supplement: Supplementary file 1 [file Data_Sheet_1.docx]

# Methods

Apoptosis assays

To further evaluate cytotoxic of SSA with the concentration of 5 and 10 μM, apoptosis was examined by Annexin V-FITC/propidium iodide (PI) (BD, USA) staining. SUM149 or MDA-MB-231 cells were seeded into 6-well plates to incubate overnight, and then were treated with different doses of SSA (0, 5, 10 μM). Twenty-four or 48 hours later, both of adherent and suspended cells in 6-well plate were collected for Annexin V-FITC/PI staining according to the instruction of manufacturer. Briefly, cells were stained with Annexin V-FITC and PI (5 μL per 1×105 cells) for 30 min at room temperature and Immediately detected by flow cytometry (BD Accuri™ C6, USA) within 1 hour.

Sup. 1 SSA does not induce apoptosis in TNBC cells. SUM149 and MDA-MB-231 cells were treated with SSA at different concentrations (0, 5 and 10 μM). (A) At 24 and 48 hours, apoptosis was analysed by Annexin V-FITC and PI staining. Right histogram is data pooled from apoptosis assays. (B) At 24 h, Capase-3 cleavages were detected by western blotting analysis. Figure represents three independent experiments.
