# Supplementary figures and images for: Saikosaponin A Inhibits Triple-Negative Breast Cancer Growth and Metastasis Through Downregulation of CXCR4
Source: Front Oncol. 2020 Jan 28;9:1487. doi: 10.3389/fonc.2019.01487 (PMC6997291; doi:10.3389/fonc.2019.01487)

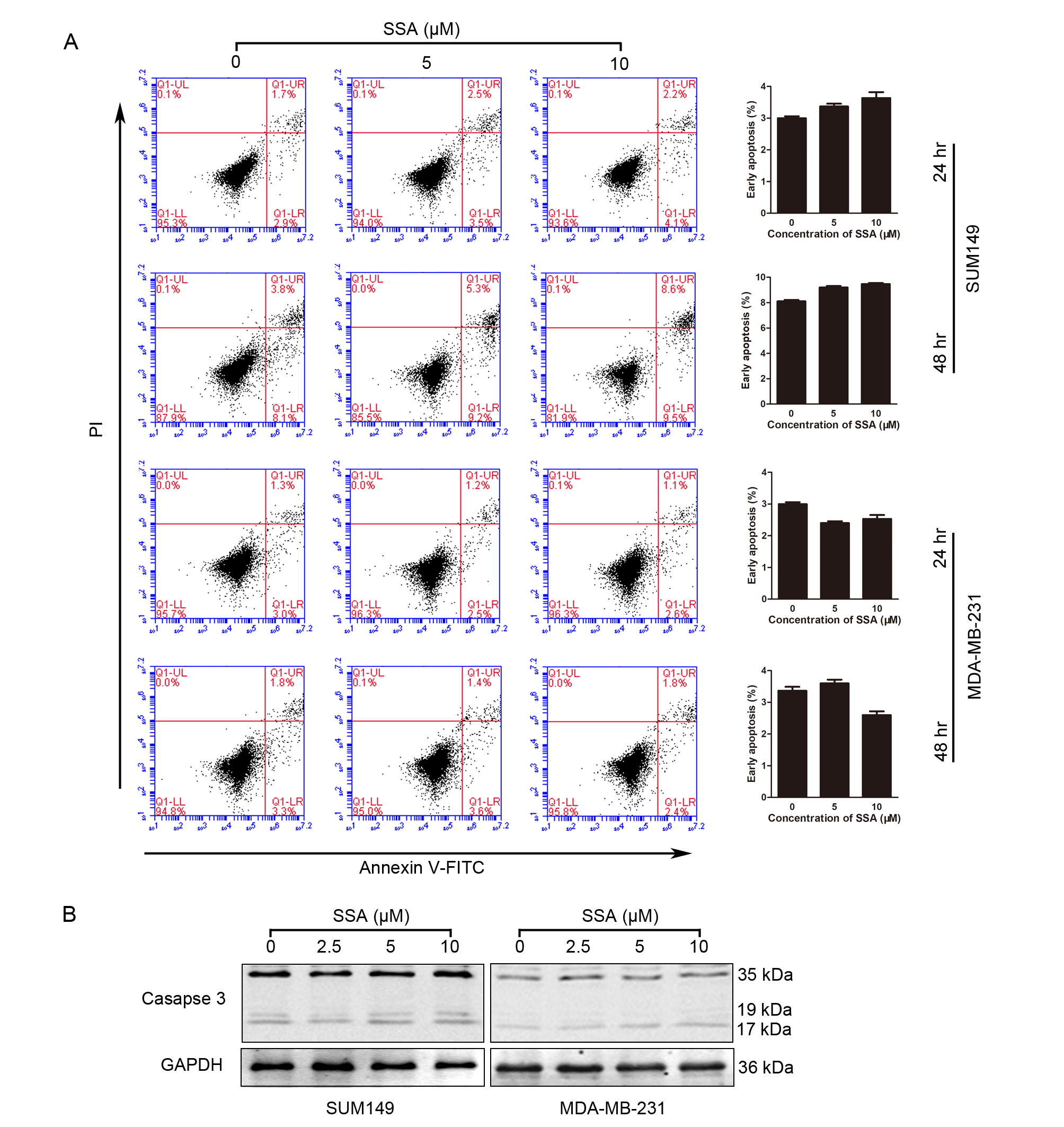

Supplement: Supplementary file 2 [file Image_1.TIF]
